# The Effect of Filtering on Signal Features of Equine sEMG Collected During Overground Locomotion in Basic Gaits

**DOI:** 10.3390/s25102962

**Published:** 2025-05-08

**Authors:** Małgorzata Domino, Marta Borowska, Elżbieta Stefanik, Natalia Domańska-Kruppa, Michał Skibniewski, Bernard Turek

**Affiliations:** 1 Department of Large Animal Diseases and Clinic, Institute of Veterinary Medicine, Warsaw University of Life Sciences, 02-787 Warsaw, Poland; elzbieta_stefanik@sggw.edu.pl (E.S.); natalia_domanska-kruppa@sggw.edu.pl (N.D.-K.); bernard_turek@sggw.edu.pl (B.T.); 2 Institute of Biomedical Engineering, Faculty of Mechanical Engineering, Białystok University of Technology, 15-351 Bialystok, Poland; 3 Department of Morphological Sciences, Institute of Veterinary Medicine, Warsaw University of Life Sciences (WULS–SGGW), 02-787 Warsaw, Poland; michal_skibniewski@sggw.edu.pl

**Keywords:** horse, muscle myoelectric activity, surface electromyography, signal processing, signal filtering, signal loss

## Abstract

In equine surface electromyography (sEMG), challenges related to the reliability and interpretability of data arise, among other factors, from methodological differences, including signal processing and analysis. The aim of this study is to demonstrate the filtering–induced changes in basic signal features in relation to the balance between signal loss and noise attenuation. Raw sEMG signals were collected from the quadriceps muscle of six horses during walk, trot, and canter and then filtered using eight filtering methods with varying cut–off frequencies (low–pass at 10 Hz, high–pass at 20 Hz and 40 Hz, and bandpass at 20–450 Hz, 40–450 Hz, 7–200 Hz, 15–500 Hz, and 30–500 Hz). For each signal variation, signal features—such as amplitude, root mean square (RMS), integrated electromyography (iEMG), median frequency (MF), and signal–to–noise ratio (SNR)—along with signal loss metrics and power spectral density (PSD), were calculated. High–pass filtering at 40 Hz and bandpass filtering at 40–450 Hz introduced significant filtering–induced changes in signal features while providing full attenuation of low–frequency noise contamination, with no observed differences in signal loss between these two methods. Other filtering methods led to only partial attenuation of low–frequency noise, resulting in lower signal loss and less consistent changes across gaits in signal features. Therefore, filtering–induced changes should be carefully considered when comparing signal features from studies using different filtering approaches. These findings may support cross-referencing in equine sEMG research related to training, rehabilitation programs, and the diagnosis of musculoskeletal diseases, and emphasize the importance of applying standardized filtering methods, particularly with a high–pass cut–off frequency set at 40 Hz.

## 1. Introduction

Surface electromyography (sEMG) is a non–invasive technique that uses surface electrodes placed on the skin over selected muscles or muscle groups to measure skeletal muscle myoelectric activity [1,2]. In equine sports applications, sEMG—unlike needle electromyography (nEMG)—can be used to monitor horses’ daily performance in non–clinical conditions [3], making it a valuable tool for advancing equine biomechanics [4] and improving training methods [5,6]. In equine medical applications, sEMG is also increasingly used for diagnosing and monitoring the treatment of orthopedic [7,8,9] and neurological diseases [10,11], as well as for assessing rehabilitation efficiency [12]. Despite the undeniable clinical utility of sEMG in horses, comparing sEMG data across studies remains challenging due to variations in signal collection, processing, and analysis methods, stating a problem with the reliability and interpretability of sEMG data in equine applications.

Challenges in comparing equine sEMG studies arise, amongst other factors, from horse preparation for sEMG [13], electrode placement [13,14], and signal processing methods [15]. One may observe that early equine sEMG studies have focused on analyzing raw signals [10,16,17,18]. Over time, the need for filtering became evident, leading to the adaptation of filtering protocols from human medicine. These included low–pass filtering at a 10 Hz cut–off frequency [19,20,21], high–pass filtering at a 20 Hz cut–off frequency [11,22], high–pass filtering at a 40 Hz cut–off frequency [23], and bandpass filtering at 7–200 Hz cut–off frequency [24,25,26,27]. It was not until 2018 that St. George et al. [15] highlighted the necessity of high–pass filtering to remove low–frequency noise caused by movement artifacts. They investigated high–pass filtering with 20 Hz, 40 Hz, 60 Hz, and 80 Hz cut–off frequencies, recommending a 40 Hz high–pass filter as optimal [15]. In 2019, St. George et al. [28] further examined sEMG signals processed with high–pass filtering at a 40 Hz cut–off frequency, reaffirming the recommendation for a 40 Hz high–pass cut–off level [15]. Subsequent studies adopted this approach, using systems with an internal bandwidth between high–pass level at 20 Hz and low–pass level at 450 Hz, followed by the use of high–pass filtering at 40 Hz [4,6,8,9]; high–pass filtering at 40 Hz combined with low–pass filtering at 450 Hz [5]; or bandpass filtering within the 40–450 Hz range [14,29].

This growing number of studies conducted in a more standardized, evidence-based manner represents a long-awaited move towards the standardization of equine sEMG. However, despite recommendations for equine sEMG advocating a 40 Hz high–pass cut–off frequency [15,28], some studies still apply alternative filtering methods, such as 15–500 Hz [30], and 30–500 Hz [31]. This may result from a limited understanding of sEMG signal filtering mechanisms and the impact of filtering on research outcomes. While it is generally acknowledged that different sEMG filtering approaches influence the resulting signal features, this effect has not yet been comprehensively documented. Therefore, in this study, eight previously used filtering methods—low–pass at 10 Hz, high–pass at 20 Hz and 40 Hz; and bandpass at 20–450 Hz, 40–450 Hz, 7–200 Hz, 15–500 Hz, and 30–500 Hz—were applied to the same sEMG signal to explore their impact on signal characteristics. We believe that a systematic report on how filtering affects sEMG signal features, and the balance between nose attenuation and residual signal, will support wider adoption of the recommended filtering method for equine sEMG. This, in turn, will promote standardization of signal processing and enhance the utility of sEMG in equine sports and medicine while facilitating better cross-referencing of previous sEMG studies.

In sEMG studies, signal filtering is essential for removing contaminations that can distort raw signal interpretation [15,32]. Both low– and high–frequency noise can contaminate the sEMG signal [32,33,34,35]. Low–frequency noise, such as power line noise, can be eliminated using a notch filter (typically at 50 Hz) [36], while noise originating from the skin-electrode interface can be eliminated using high–pass filtering [34]. An example of these last noises is the movement artifacts, which typically occupy a spectral range between 0 and 20 Hz [32,37]. In human sEMG processing, low–frequency noise is commonly removed using a high–pass filtering at a 20 Hz cut–off frequency [32], while in equine subjects, greater body weight, muscle mass, gait speed, and gait characteristics lead to a broader spectral range occupied by the movement artifacts [15] and a higher cut–off frequency—specifically 40 Hz [15,28]. High–frequency noise, originating from the inter-electrode distance (IED), tissue properties, and electrode placement [33,35], is typically attenuated using low–pass filtering. This type of contamination becomes more pronounced when electrodes are positioned near tendons rather than over the muscle belly, making amplitude-based features highly placement–sensitive [33,35]. In human sEMG studies, high–frequency noise is typically attenuated using low–pass filtering with a cut–off around 400–450 Hz [5,14,29,32]. A separate low-pass filtering at 10 Hz is used post-terification to extract the signal envelope [19]. This approach differs entirely from filtering aimed at noise attenuation and is included here to distinguish clearly between these two signal processing objectives.

It is important to note that any sEMG signal attenuation inevitably results in some degree of signal loss, which depends on the selected cut-off frequencies [32,37]. This can be quantified using signal loss metrics, reflecting a reduction in both desired signal and noise components [15,32]. Reducing the desired signal component introduces filtering-induced changes to the signal features, which should be considered when cross-referencing equine sEMG studies that use different filtering methods. While filtering alters signal features, such changes are acceptable if noise is effectively minimized. Therefore, power spectral density analysis can evaluate the balance between noise attenuation and signal preservation [15,38]. However, beyond one equine sEMG study analyzing signal loss and power spectral density across various high-pass settings [15], the broader impact of filtering methods on signal characteristics remains unclear. Consequently, it is unclear which study results can be directly compared and which require adjustments based on the applied signal filtering.

Given that different methods of sEMG signal filtering attenuate both the desired signal and noise in various ways [15,32], and recognizing the existing gap in the literature regarding how various filtering approaches influence signal features, we hypothesize that signal features extracted from a representative muscle vary depending on the filtering method applied across three basic gaits. Therefore, the aim of this study is to demonstrate the filtering-induced changes in basic signal features in relation to the balance between signal loss and noise attenuation—both for previously used noise attenuation filtering methods (high-pass at 20 Hz and 40 Hz, and bandpass at 20–450 Hz, 40–450 Hz, 7–200 Hz, 15–500 Hz, and 30–500 Hz) and, for contrast, specific low-pass filtering at 10 Hz. We believe these findings can support improved cross-referencing in equine sEMG studies, particularly those related to biomechanics, rehabilitation programs, and the diagnosis of musculoskeletal disorders.

## 2. Materials and Methods

### 2.1. sEMG Signal Collection

The sEMG signal was collected from six middle-aged Konik Polski horses (four females, two males; median age: 8.5 years, range: 6–12 years; median body mass: 435 kg, range: 420–450 kg; median height at withers: 135 cm, range: 135–145 cm). All horses were deemed sound by an experienced veterinarian and had no known history of neurological disorders. The horses were fed individually calculated rations of hay and concentrate, divided into three meals per day, with constant access to a mineral salt block and fresh water. The horses were at the same preliminary training level (working under saddle and correctly performing all three basic gaits: walk, trot, and canter), were regularly exercised, had daily turnout with access to a sandy paddock, and were housed under uniform environmental conditions in box stalls within the same stable.

**Figure 1:**
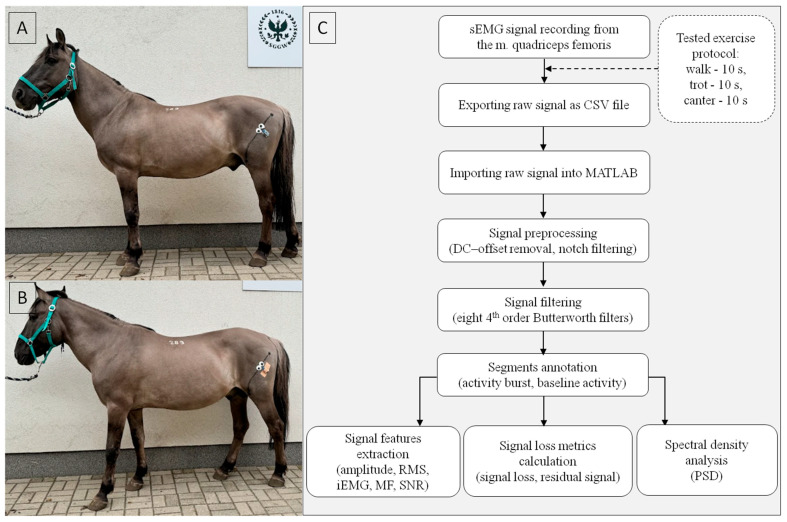
Study design diagram. (**A**) Placement of surface electrodes and sensor in relation to black line between anatomical landmarks for m. quadriceps femoris. (**B**) Stabilization of sensor position using kinesiology tape. (**C**) Flow diagram of the sEMG signal processing. Filtering methods: low-pass 10 Hz, high-pass 20 Hz, high-pass 40 Hz, bandpass 20–450 Hz, bandpass 40–450 Hz, bandpass 7–200 Hz, bandpass 15–500 Hz, and bandpass 30–500 Hz. Signal features: amplitude, root mean square (RMS), integrated electromyography (iEMG), median frequency (MF),signal-to-noise ratio (SNR). Spectral density analysis: power spectral density (PSD). Tested exercise protocol: overground, in hand, in a straight line, each gait per 10 s.

On the day of signal collection, the horse’s lateral right hind limb was clipped from the hip joint to the knee joint area. The clipper blade set at 0.8 mm was used to remove hair from electrode sites. Then the clipped area was cleaned with 80% ethanol. Specific anatomical landmarks for optimal electrode placement on the m. quadriceps femoris, specifically the m. vastus lateralis, were identified by palpation of the cranial part of the greater trochanter of the femur and the tibial crest [14]. A line connecting these two landmarks was drawn, and two surface electrodes (pre-gelled Ag/AgCl; 4 cm diameter; self-adhesive; 4 cm IED; 270Bx, Noraxon, Scottsdale, AZ, USA) were placed at 40–50% of the proximal section of this line [14]. Following SENIAM guidelines [39], the electrodes were positioned next to each other, aligned with the muscle fiber direction. The m. quadriceps femoris location, its attachment points to bone landmarks, and muscle fiber orientation were confirmed ultrasonographically using a SonoSite Edge II ultrasound machine (Fujifilm, Valhalla, NY, USA) with a 2–9 MHz convex probe (Fujifilm, Valhalla, NY, USA). Ultrasound examination starting from the greater trochanter of the femur revealed that, in the proximal region, the m. quadriceps femoris was overlain by the m. tensor fasciae latae, m. biceps femoris, and m. gluteal muscles. The examination identified the distal boundary of this overlay, and electrodes were placed where the m. quadriceps femoris was more superficial and no longer covered by adjacent muscles. A drop of conductive gel (Indiba, Las Rozas, Spain) was applied between each electrode and the skin, using the smallest possible volume to cover the entire conductive surface of the electrode without reaching its adhesive surface. The electrodes were connected to a wireless sEMG sensor (DTS, Noraxon, Scottsdale, AZ, USA) in a bipolar configuration (Figure 1A). The sensor was stuck to the skin with dedicated self-adhesive stickers #874E (Noraxon, Scottsdale, AZ, USA) and further stabilized using Pino Tape Sport kinesiology tape (Pino, Ahrensburg, Germany) (Figure 1B). The adhesion of the electrodes and kinesiology tape was enhanced with a thin layer of Rudaspray medical glue (Nobamed, Bielefeld, Germany). The glue was sprayed onto the entire surface of the electrodes and tapes immediately before attaching them to the skin.

The sEMG signal was recorded using the Noraxon Ultium electromyograph (Noraxon, Scottsdale, AZ, USA) with a sampling frequency of 2000 Hz and internal bandwidth between 10 and 500 Hz. Data were stored using MR software version 3.18.98 (Noraxon, Scottsdale, AZ, USA). The sEMG signal was collected during the overground tested exercise. Horses were led in hand in a straight line, first at a walk, then at a trot, and finally at a canter. Prior to recording day, horses were trained to canter in hand on voice command. Each tested exercise and consequently each sEMG signal recording lasted at least 10 s. Before starting the measurement, electrode–skin impedance levels were tested using thebuilt-in function of MR software version 3.18.98 (Noraxon, Scottsdale, AZ, USA) to ensure proper electrode adhesion. During each measurement, each horse completed at least 8 strides at a walk, 16 strides at trot, and 20 strides at canter. Each measurement was repeated three times, and the best record free of artifacts and rare electrode contact loss was selected for further analysis.

### 2.2. sEMG Signal Processing

Data stored in MR software version 3.18.98 (Noraxon, Scottsdale, AZ, USA) were extracted as CSV files and imported into MATLAB software version R2024b (MathWorks, Natick, MA, USA) as raw signal. The raw signal was preprocessed by DC-offset removal, and notch filtering to eliminate the power line noise, specifically targeting the 50 Hz frequency [36]. No additional normalization was applied. Subsequently, the signal was filtered with a 4th order Butterworth filter using the following cut-off frequencies separately: (1) low-pass filtering at 10 Hz cut-off frequency, (2) high-pass filtering at 20 Hz cut-off frequency, (3) high-pass filtering at 40 Hz cut-off frequency, (4) bandpass filtering at 20–450 Hz cut-off frequency, (5) bandpass filtering at 40–450 Hz cut-off frequency, (6) bandpass filtering at 7–200 Hz cut-off frequency, (7) bandpass filtering at 15–500 Hz cut-off frequency, and (8) bandpass filtering at 30–500 Hz cut-off frequency. The low-pass filtered signal was rectified. As a result, nine variations of the same sEMG signals were obtained and marked as raw signals, and eight filtered signals: low-pass 10 Hz, high-pass 20 Hz, high-pass 40 Hz, bandpass 20–450 Hz, bandpass 40–450 Hz, bandpass 7–200 Hz, bandpass 15–500 Hz, and bandpass 30–500 Hz, respectively. Thus, eight signal variations refer to the same sEMG signal but with different filters applied. Each signal variation was analyzed using the same protocol as shown in Figure 1C.

### 2.3. sEMG Signal Features Extraction

Following our preliminary study [40], for each signal variation, eight burst segments (activity burst) and eight noise segments (no activity burst referred to as baseline activity) were manually selected by an experienced evaluator, corresponding to the eight strides per gait. The number of strides was standardized to eight based on the lowest number of strides collected in walk. Each segment was marked between two time cursors annotated on the raw signal and then replicated after filtering. Thus each burst segment was extracted as a separate region of interest (ROI) maintaining the same duration. To ensure a systematic approach, the criteria for burst identification were: the series of signal spikes with the value above |10 mV| and duration greater than 0.1 s. The duration of each ROI was determined by its individual characteristics, so each ROI does not contain the same number of samples. For each signal variation, eight burst ROIs and eight noise ROIs were saved and exported as separate MATLAB files.

For each burst ROI, the following signal features were extracted using the Signal Analyzer application in MATLAB [41]: amplitude, root mean square (RMS), integrated electromyography (iEMG), and median frequency (MF). For each pair of burst ROI and following noise ROI signal-to-noise ratio (SNR) was calculated.

Amplitude was extracted as the maximum absolute value of the signal and is expressed in [mV]. RMS was extracted as the mean square of signal values used to determine the magnitude of a signal variation, using Formula (1), and is expressed in [mV]:

(1) $$RMS=\sqrt{\frac{1}{N}\sum_{n=1}^{N}{\left(x\left(n\right)\right)}^{2}}$$

where *N* is the length of signal *x*. iEMG was extracted as the sum of absolute signal values divided by length of the signal, using Formula (2), and is expressed in [mV×s,]:

(2) $$iEMG=\frac{1}{N}\sum_{n=1}^{N}\left|x(n)\right|$$

MF was extracted as a frequency value at which the sEMG power spectrum is divided into two regions with an equal integrated power after the application of a Discrete Fourier Transform (DFT) using a Formula (3):

(3) $$X_{k}=\sum_{n=0}^{N-1}x\left(n\right)e^{\frac{-i2\pi }{N}kn}$$

where *i* is the imaginary unit. MF was calculated using a Formula (4) and is expressed in [Hz]:

(4) $$\sum_{j=1}^{MF}P_{j}=\sum_{j=MF}^{M}P_{j}=\frac{1}{2}\sum_{j=1}^{M}P_{j}$$

where $P_{j}=\frac{1}{N^{2}}{\left|X_{k}\right|}^{2}$ is the signal power spectrum at the frequency bin *j* and M is the length of frequency bin defined as the next power of 2 from the length of signal in the time domain. SNR was extracted as the ratio of the signal (burst ROIs) to the following background signal (noise ROIs), approximated using Formula (5), and is expressed in [dB]:

(5) $${SNR}_{i}=10\cdot \log10\frac{{RMS\left(burst\;{ROIs}_{i}\right)}^{2}}{{RMS\left(noise\;{ROIs}_{i}\right)}^{2}},$$

where i is the $i^{th}$ extracted segment of signal.

### 2.4. sEMG Signal Loss Metrics Calculation

For each signal variation represented filtered signal (low-pass 10 Hz, high-pass 20 Hz, high-pass 40 Hz, bandpass 20–450 Hz, bandpass 40–450 Hz, bandpass 7–200 Hz, bandpass 15–500 Hz, and bandpass 30–500 Hz), signal loss and residual signal were calculated following previous studies [15,40] and using the RMS values and Formulas (6) and (7), respectively:

(6) $$Signal\;Loss\left(\%\right)=\frac{\left({RMS}_{full}-{RMS}_{filter}\right)}{{RMS}_{full}}$$

(7) $$Residual\;Signal\left(\%\right)=\frac{{RMS}_{filter}}{{RMS}_{full}}$$

where RMS_full_ is the RMS value of the raw signal and RMS_filter_ is the RMS value of the filtered signal. Both signal loss metrics are expressed in [%].

### 2.5. Power Spectral Density Calculation

Similarly, for each signal variation the Fast Fourier Transformation (FFT) was applied in MATLAB and the power spectral density (PSD) was calculated using a Hanning window following St. George et al. [15]. FFT length, window length, and window overlap were estimated based on mean burst duration, which was changed across gaits: walk; trot, and canter. The mean ± SD burst duration was 0.63 ± 0.11 [s] in walk, 0.41 ± 0.05 [s] in trot, and 0.21 ± 0.02 [s] in canter. Thus the adjusted FFT length: 0.25; window length: 0.125; and overlap length: 0.0625 based on average duration across gaits, were used. Finally, PSD was calculated using function (8), and expressed in [mV^2^/Hz]:

(8) $$\left[psd,f\right]=pwelch(A\_filtr,hanning(window\_length),overlap\_length,fft\_length,fs)$$

where: window_length = round(0.125 × duration × fs), overlap_length = round(0.0625 × duration × fs), fft_length = round(0.25 × duration × fs); duration = gait_durations.(gait), and gait_durations = struct(‘walk’, 0.63, ‘trot’, 0.41, ‘canter’, 0.21).

### 2.6. Statistical Analysis

Statistical analysis was performed using Prism version 6 (GraphPad Software Inc., San Diego, CA, USA). The dataset was prepared as a series of data representing each signal feature, signal loss metric, and PSD, containing 144 realizations (8 burst segments × 6 horses × 3 gaits). Each data series was tested for normality using the Shapiro–Wilk test. Since not all data series followed a normal distribution, numerical data were presented as the median and quartiles (Q1, Q3).

Data series of signal features were compared between raw signal and filtered signals, except low-pass filtering at 10 Hz, which was compared separately. The raw signal and seven filtered signals were compared as paired data (the mean of each column representing filtered signals with the mean of a control column representing raw signal) within each gait—walk, trot, and canter—separately. Data series of signal loss metrics and PSD were compared across seven signal variations, except low-pass filtering at 10 Hz, which was compared separately. The raw signal and seven filtered signals were compared also as paired data within each gait—walk, trot, and canter—separately; however, the mean of each column was compared with the mean of each other column, rather than the mean of a control column. When all data series in a compared dataset were normally distributed, a Repeated Measures ANOVA was used. If at least one data series in a compared dataset was not normally distributed, a Friedman test was performed. When significant differences were detected, post-hoc Tukey’s multiple comparisons test was applied for normally distributed data, while the post-hoc Dunn’s multiple comparisons test was used for non-normally distributed data.

Data series of signal features and PSD after low-pass filtering at 10 Hz were compared with the raw signal using a paired *t* test when both data series were normally distributed or a Wilcoxon matched-pairs signed rank test when at least one data series was not normally distributed. Statistical significance was set at *p* < 0.05.

## 3. Results

### 3.1. sEMG Signal Characteristics

The values of sEMG signal features recorded from the m. quadriceps femoris varied significantly between the raw signal and its filtered variations, as shown in Table 1 for walk, Table 2 for trot, and Table 3 for canter.

Amplitude, RMS, and MF were significantly lower (*p* < 0.0001) than in the raw signal after low-pass filtering at 10 Hz across all gaits; while SNR was significantly lower in trot and canter (*p* < 0.0001). Amplitude, RMS, and iEMG were significantly lower (*p* < 0.0001) than in the raw signal, while MF and SNR were significantly higher (*p* ≤ 0.0004) consistently across all gaits after high-pass filtering at 40 Hz, bandpass filtering at 40–450 Hz, and bandpass filtering at 30–500 Hz. RMS and iEMG, but not amplitude, were significantly lower (*p* < 0.0001) than in the raw signal consistently across all gaits after high-pass filtering at 20 Hz, bandpass filtering at 20–450 Hz, and bandpass filtering at 15–500 Hz. MF and SNR were significantly higher lower (*p* ≤ 0.0004) than in the raw signal consistently across all gaits after high–pass filtering at 20 Hz and bandpass filtering at 20–450 Hz, whereas only SNR, but not MF, was significantly higher (*p* ≤ 0.0004) consistently across all gaits after bandpass filtering at 15–500 Hz. After bandpass filtering at 7–200 Hz no increase or decrease in the signal features were noted that were consistent across all gaits. For this filtering, amplitude and iEMG were significantly lower (*p* < 0.0001) than in the raw signal during walk and canter, but not during trot; while RMS was significantly lower (*p* < 0.0001) than in the raw signal during trot and canter, but not during walk. No significant differences (*p* ≥ 0.05) were found between the raw and filtered signal for iEMG after low-pass filtering at 10 Hz; amplitude after high-pass filtering at 20 Hz, bandpass filtering at 20–450 Hz, and bandpass filtering at 15–500 Hz; MF after bandpass filtering at 7–200 Hz and 15–500 Hz; as well as SNR after bandpass filtering at 7–200 Hz.

**Figure 2:**
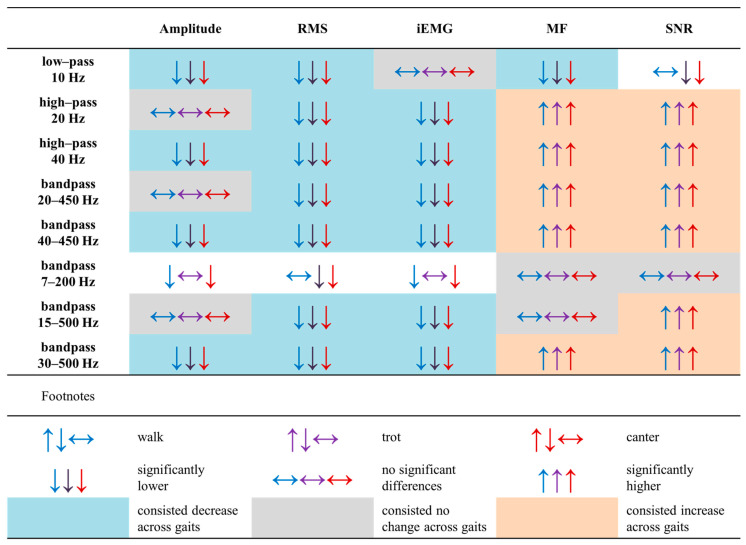
A summary of the effect of the different filtering methods on each sEMG signal feature studied across three basic gaits: walk, trot, and canter. Significant differences (*p* ≤ 0.0004) of signal feature in relation to the raw signal (significantly lower, no significant differences, or significantly higher) are marked by arrows for each gait (walk—blue arrow; trot—purple arrow; canter—red arrow) and highlighted at background of the appropriate color when the change is consistent across gaits. Signal features: amplitude, root mean square (RMS), integrated electromyography (iEMG), median frequency (MF), signal-to-noise ratio (SNR).

**Figure 3:**
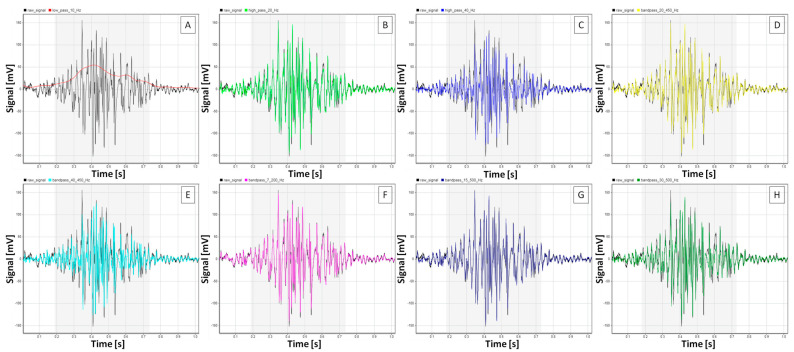
Representative burst segments of sEMG signals from the m. quadriceps femoris of n = 1 horse during walk. Burst segment of filtered signals (colored lines) presented on the background of the raw signal (black line) after (**A**) low-pass filtering at 10 Hz, (**B**) high-pass filtering at 20 Hz, (**C**) high-pass filtering at 40 Hz, (**D**) bandpass filtering at 20–450 Hz, (**E**) bandpass filtering at 40–450 Hz, (**F**) bandpass filtering at 7–200 Hz, (**G**) bandpass filtering at 15–500 Hz, and (**H**) bandpass filtering at 30–500 Hz. Gray frames mark burst segment annotations.

**Figure 4:**
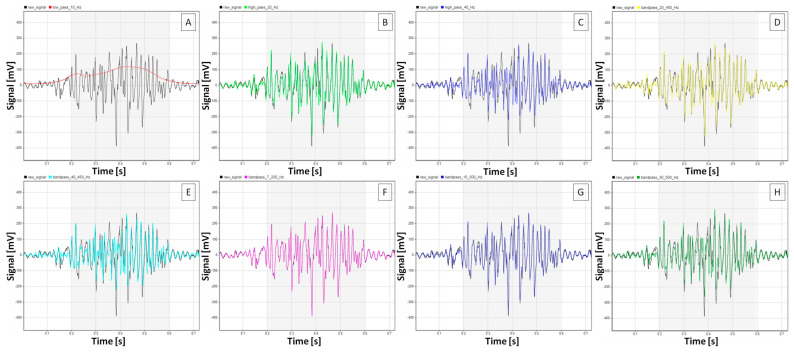
Representative burst segments of sEMG signals from the m. quadriceps femoris of n = 1 horse during trot. Burst segment of filtered signals (colored lines) presented on the background of the raw signal (black line) after (**A**) low-pass filtering at 10 Hz, (**B**) high-pass filtering at 20 Hz, (**C**) high-pass filtering at 40 Hz, (**D**) bandpass filtering at 20–450 Hz, (**E**) bandpass filtering at 40–450 Hz, (**F**) bandpass filtering at 7–200 Hz, (**G**) bandpass filtering at 15–500 Hz, and (**H**) bandpass filtering at 30–500 Hz. Gray frames mark burst segment annotations.

**Figure 5:**
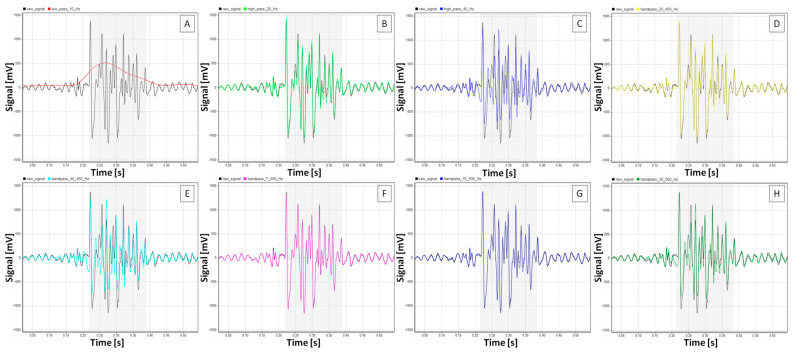
Representative burst segments of sEMG signals from the m. quadriceps femoris of n = 1 horse during canter. Burst segment of filtered signals (colored lines) presented on the background of the raw signal (black line) after (**A**) low-pass filtering at 10 Hz, (**B**) high-pass filtering at 20 Hz, (**C**) high-pass filtering at 40 Hz, (**D**) bandpass filtering at 20–450 Hz, (**E**) bandpass filtering at 40–450 Hz, (**F**) bandpass filtering at 7–200 Hz, (**G**) bandpass filtering at 15–500 Hz, and (**H**) bandpass filtering at 30–500 Hz. Gray frames mark burst segment annotations.

A summary of the effect of the different filtering methods on each sEMG signal feature studied here is presented in Figure 2. In contrast, representative sEMG signals before and after applying each filtering method are presented in Figure 3 for walk, Figure 4 for trot, and Figure 5 for canter.

### 3.2. sEMG Signal Loss Metrics and Power Spectral Density

The values of signal loss metrics and PSD for the sEMG signal recorded from the m. quadriceps also significantly varied among the filtered variations, as shown in Table 4 for walk, Table 5 for trot, and Table 6 for canter.

**Figure 6:**
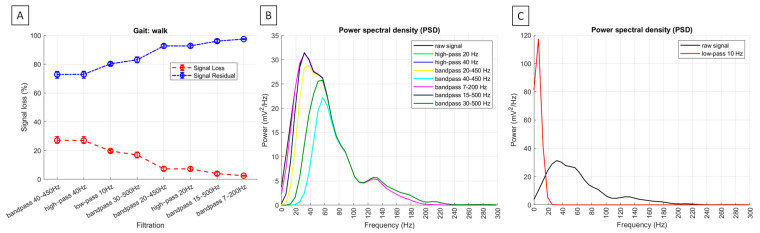
Signal loss metrics and power spectral density (PSD) of sEMG signal from the m. quadriceps femoris during walk. (**A**) Median and range (Q1; Q3) signal loss (%) (red line) and residual signal (%) (blue line) among the signal variations across n = 6 horses. Representative PSD for burst segment of n = 1 horse. PSD is presented after filtering using (**B**) noise attenuation methods and (**C**) specific low–pass filtering at 10 Hz. After filtering, PSD is displayed by colored lines, while a black line represents the raw signal.

**Figure 7:**
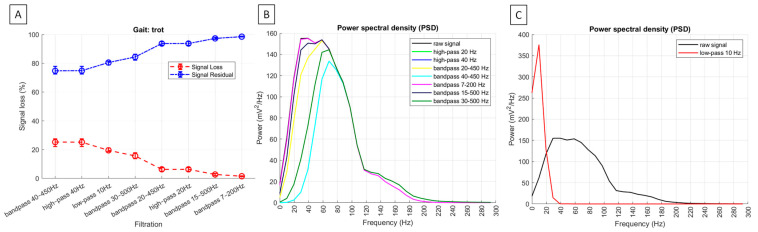
Signal loss metrics and power spectral density (PSD) of sEMG signal from the m. quadriceps femoris during trot. (**A**) Median and range (Q1; Q3) signal loss (%) (red line) and residual signal (%) (blue line) among the signal variations across n = 6 horses. Representative PSD for burst segment of n = 1 horse. PSD is presented after filtering using (**B**) noise attenuation methods and (**C**) specific low–pass filtering at 10 Hz. After filtering, PSD is displayed by colored lines, while a black line represents the raw signal.

**Figure 8:**
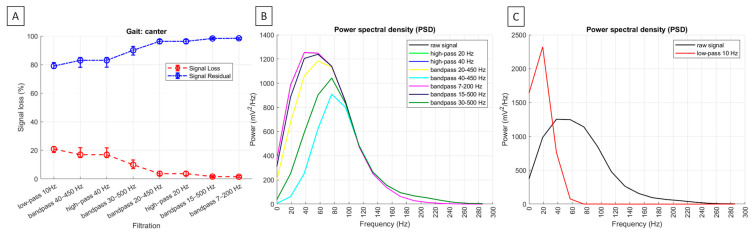
Signal loss metrics and power spectral density (PSD) of sEMG signal from the m. quadriceps femoris during canter. (**A**) Median and range (Q1; Q3) signal loss (%) (red line) and residual signal (%) (blue line) among the signal variations across n = 6 horses. Representative PSD for burst segment of n = 1 horse. PSD is presented after filtering (**B**) noise attenuation methods and (**C**) specific low–pass filtering at 10 Hz. After filtering, PSD is displayed by colored lines, while a black line represents the raw signal.

Across all gaits, the significantly highest (*p* < 0.0001) signal loss was observed after high-pass filtering at 40 Hz and bandpass filtering at 40–450 Hz, with no significant difference between these two filtering methods. Significantly lower signal loss (*p* < 0.0001) was found after bandpass filtering at 30–500 Hz during trot and canter. Even significantly lower signal loss (*p* < 0.0001) was noted after bandpass filtering at 30–500 Hz during walk, as well as after high-pass filtering at 20 Hz and bandpass filtering at 20–450 Hz across all gaits, with no significant difference between the latest two filtering methods. The significantly lowest signal loss (*p* < 0.0001) was recorded after bandpass filtering at 15–500 Hz and 7–200 Hz, with significant differences between these two filtering methods during walk but no significant differences during trot and canter. Since residual signal is the inverse of signal loss among signal loss metrics, it followed the same pattern in the opposite direction. Signal loss metrics and representative PSDs for burst segments are shown in Figure 6 for walk, Figure 7 for trot, and Figure 8 for canter.

Across all gaits after, the PSD was significantly higher after low-pass filtering at 10 Hz than in the raw signal (*p* < 0.0001). When considering all other filtering methods, the PSD was significantly the highest in the raw signal and after bandpass filtering at 7–200 Hz and 15–500 Hz, significantly lower after high-pass filtering at 20 Hz and bandpass filtering at 20–450 Hz, even significantly lower after bandpass filtering at 30–500 Hz, and significantly the lowest after high-pass filtering at 40 Hz and bandpass filtering at 40–450 Hz (*p* < 0.0001). However, the visual inspection of PSD plots showed the low-frequency noise components between 0 and 20 Hz, which was fully attenuated after high-pass filtering at 40 Hz and bandpass filtering at 40–450 Hz during walk (Figure 6B) and trot (Figure 7B), almost fully attenuated after filtering using these two methods during canter (Figure 8B). The visual inspection of PSD plots showed also that the low-frequency noise components were partially attenuated after applying all other filtering methods (Figure 6B, Figure 7B and Figure 8B), except for the low-pass filtering at 10 Hz, where the PSD visually appear as an increase or ‘amplification’ of the low-frequency band in the frequency domain (Figure 6C, Figure 7C and Figure 8C).

## 4. Discussion

This study intends to support the cross–referencing in equine sEMG studies, therefore the signal features—including amplitude, RMS, iEMG, MF, and SNR—were chosen from the broad range of signal features available for EMG signal analysis [41], based on their relevance in previous equine sEMG studies. The obtained results support the hypothesis that signal features recorded from the m. quadriceps femoris vary significantly depending on the filtering method applied across the three basic gaits. However, these differences should be interpreted in the context of the balance between signal loss and noise attenuation, which represents a significant advancement of this study compared to our preliminary work [40].

### 4.1. Effect of Noise Attenuation Filtering Methods on Basic sEMG Signal Features

Across all gaits, the least amount of signal loss was observed for bandpass filtering at 7–200 Hz. With this filtering method, no consistent changes in MF and SNR were noted across gaits, while amplitude and amplitude–related signal features were, in some gaits, significantly lower than in the raw signal. This suggests that bandpass filtering at 7–200 Hz attenuates the fewest signal components—both desired signal and noise—and that the resulting signal is the most similar to the raw signal. However, also the PSD plots of the raw signal and signal after bandpass filtering at 7–200 Hz were nearly overlapping—especially within the spectral range between 0 and 20 Hz, which is typically occupied by the movement artifacts [32,37]—indicating that noise component attenuation was the least effective. It should also be noted that the internal bandwidth of the system used in this study was 10–500 Hz; therefore, the effective bandwidth of the signal filtering at 7–200 Hz was even narrower, and the noise attenuation efficiency was likely slightly lower.

For bandpass filtering at 15–500 Hz, signal loss was significantly higher than that observed after bandpass filtering at 7–200 Hz during walk, but comparable in trot and canter. With this filtering method, no consistent changes in amplitude and MF were noted across gaits, while amplitude–related signal features were significantly lower than in the raw signal, and SNR was significantly higher. This suggests that bandpass filtering at 15–500 Hz causes slightly greater attenuation of both signal components, sufficient to introduce consistent modifications across gaits to the desired signal, as reflected in the signal features changes. However, this filtering method remains insufficient to effectively attenuate the noise component in the 0–20 Hz range, as indicated by only partial attenuation visible in the PSD plots.

For high–pass filtering at 20 Hz and bandpass filtering at 20–450 Hz, the signal loss was significantly higher than that observed after bandpass filtering at 7–200 Hz and 15–500 Hz. With these filtering methods, no consistent changes in amplitude were noted across gaits, while amplitude–related signal features were significantly lower than in the raw signal, and both MF and SNR were significantly higher. This suggests that filtering with a high–pass cut–off set at 20 Hz causes attenuation of signal components, sufficient to introduce more pronounced changes in signal features. However, these filtering methods still remain insufficient to effectively attenuate the noise component in the 0–20 Hz range, as indicated by only partial attenuation visible in the PSD plots.

For high–pass filtering at 40 Hz and bandpass filtering at 40–450 Hz and 30–500 Hz, signal loss was significantly higher than that observed with other noise attenuation filtering methods. During walk, no significant differences in signal loss were found between filtering with high–pass cut–off set at 30 Hz and 40 Hz; however, during trot and canter, signal loss was significantly higher for high–pass filtering at 40 Hz and bandpass filtering at 40–450 Hz. With all three filtering methods, amplitude and amplitude–related signal features were consistently across gaits and significantly lower than in the raw signal, while both MF and SNR were significantly higher. This suggests that filtering with high–pass cut–off set at 30 Hz and 40 Hz attenuates signal components sufficiently to introduce even more pronounced changes in signal features. However, bandpass filtering at 30–500 Hz still remains insufficient to effectively attenuate the noise component in the 0–20 Hz range; whereas both high–pass filtering at 40 Hz and bandpass filtering at 40–450 Hz result in almost fully attenuation during canter and full attenuation during walk and trot, as shown in the PSD plots.

### 4.2. Effect of Specific Low–Pass Filtering at 10 Hz on Basic sEMG Signal Features

One may observe that the specific low–pass filtering at 10 Hz has a different effect on the sEMG signal compared to noise attenuation filtering methods, which is particularly evident in the representative sEMG signals plots. This specific filtering method was included in this study because it has been previously employed in equine sEMG studies [19,20,21,42,43,44], and the differences between low–pass filtering at 10 Hz and noise attenuation filtering methods warrant emphasis.

Low–pass filtering is typically used to compute the envelope of rectified sEMG data [19]. Firstly, rectification convert negative values to positive ones [45]; then, low–pass filtering attenuates or removes the high frequency components of the sEMG signal [46]. Envelope computed in such a way, is used when data from different subjects are averaged to obtain a representative activation profile [45]. However, this processing results in approximately a 20% signal loss, along with district changes in signal features—a significant decrease in amplitude, RMS, and MF, consistent across gaits, and no significant differences in iEMG.

Interestingly, an inspection of the signal’s power spectrum reveals not attenuation, but what visually appears to be an increase or ‘amplification’ in the 0–20 Hz range in the frequency domain. Since low–pass filtering attenuates or removes the high–frequency components, the signal contains less overall power after filtering [46]. However, the proportion of energy in the low–frequency range becomes relatively larger, especially when viewed on a log–log PSD plot, which is scaled relative to the rest of the power spectrum [38]—now significantly reduced due to filtering. Therefore, this visual effect of ‘amplification’ should be understood as relative rather than absolute.

### 4.3. Recommendations for the Comparison of Equine sEMG Signal Features

The results of this study underscores the need for standardization of equine sEMG processing to enhance the comparability and interpretability of sEMG data, particularly in applications related to equine sport and medicine. Currently, the recommended standard for equine sEMG signal processing involves high–pass filtering with a cut–off frequency of 40 Hz [15,28], which is supported by the results of this study.

This recommendation has since been implemented in three ways: (1) as standalone high–pass filtering at 40 Hz [4,6,8,9]; (2) as a combination of high–pass filtering at 40 Hz and low–pass filtering at 450 Hz [5]; and (3) as bandpass filtering at 40–450 Hz [14,29]. The first approach was used to deepen understanding of overground training during canter [4,6] and jumping [4] by analyzing amplitude [4,6]; as well as to expand medical applications by analyzing the amplitude envelope during overground walking and trotting for lameness diagnosis [8,9]. The second approach was applied to analyze muscles activity during treadmill training following β–Hydroxy β–methylbutyrate supplementation, focusing on iEMG and MF [5]. The third approach was employed to study muscles activity during treadmill training after Clenbuterol administration, analyzing envelope of amplitude and MF [29]; and to optimize electrode placement based on muscles activity recorded during treadmill trotting, analyzing SNR and coefficient of variation (CoV) [14]. Given that no differences in signal loss were found across all gaits between high–pass filtering at 40 Hz and bandpass filtering at 40–450 Hz, and considering the near–overlapping PSD plots of these two signal variations, it is expected that the signal features values reported in these studies will be similar. Therefore, results from studies using the same design and signal processing methods appear to be fully comparable.

Similarly, no differences in signal loss were found across all gaits between high–pass filtering at 20 Hz and bandpass filtering at 20–450 Hz, and also PSD plots for these two filtering methods were nearly overlapping. Therefore, from a signal processing perspective, the sEMG signals were recorded during treadmill training at walk and trot [22], and during the overground walk and trot in context of diagnosing shivering [11]—both of which used high–pass filtering at 20 Hz—can be considered comparable. On the other hand, although the raw signals recorded from the m. quadriceps femoris during treadmill trotting by Crook et al. [22] and Smit et al. [14] may be similar, the filtering methods differ: Crook et al. applied high–pass filtering at 20 Hz [22], while Smit et al. used bandpass filtering at 40–450 Hz [14]. Since these methods resulted in significantly different signal loss, the filtered signals and their corresponding features may differ. Likewise, lameness was assessed during walk and trot in the context of shivering using high–pass filtering at 20 Hz [11], and in other studies using high–pass filtering at 40 Hz [8,9]. In these cases, direct comparison of signal features is also challenging due to differences in filtering methods. Even between two similar studies on fatigue during treadmill cantering [30,31], which both analyzed iEMG and MF, comparisons are complicated by the use of different filtering methods: bandpass filtering at 15–500 Hz in one [30] and 30–500 Hz in the other [31]. Notably, during canter, signal loss differed significantly between these two filtering methods. Moreover, bandpass filtering at 15–500 Hz showed no change in MF relative to the raw signal, while bandpass filtering at 30–500 Hz resulted in a higher MF compared to the raw signal. Thus, even seemingly comparable studies require careful consideration of filtering–induced changes in signal features.

When comparing sEMG signals, additional factors must be considered beyond filtering methods, including differences in the signal features analyzed, the muscles examined, electrode placement, IED, and the sEMG system used. Even with similar study designs—such as recording sEMG signals from the m. extensor carpi radialis during treadmill trotting followed by bandpass filtering at 40–450 Hz [14,29]—direct comparison of signal features is not possible when the analyzed features differ. For instance, one study extracted the envelope of amplitude and MF [29], while the other analyzed SNR and CoV [14]. Moreover, amplitude and amplitude-dependent features are sensitive to electrode placement. These values tend to increase when electrodes are positioned closer to the tendon rather than directly over the muscle belly [33]. In the present study, electrodes were placed within the common safe zone, considered optimal at 45–50% of the proximal segment of the line between the greater trochanter of the femur and the tibial crest [14]. This placement is similar to other studies on the m. quadriceps femoris, which describe the electrode position as the midpoint of the muscle belly [16,22]. Previous studies reported varying IEDs: 1.5 cm [22], 2.4 cm [19], and 2.5 cm [16], whereas the present study used an IED of 4.0 cm. It is well established that smaller IEDs capture higher–frequency components, while larger IEDs capture lower–frequency components of the signal. Additionally, smaller IEDs result in lower amplitude due to fewer motor units being detected, whereas larger IEDs yield higher amplitudes because more motor units are recorded [32,47,48]. Given these and other methodological factors that influence sEMG signal features, it is essential to strive for further standardization of equine sEMG acquisition and processing to improve comparability and interpretability across studies.

### 4.4. Limitations

It should be noted that this study was designed with a uniform group of middle–aged horses of the same breed and training level. Therefore, the generalizability of the results is limited. Including a larger and more diverse sample—incorporating horses of different breeds, ages, and training levels, as well as examining a wider range of muscles—would improve the generalizability of the findings. Due to the complexity and challenges associated with performing sEMG recordings in horses, study groups are often small and heterogeneous. For instance, the study on optimal electrode placement for measuring equine myoelectric activity [14] and our preliminary study on the effect of three filtering methods on signal features from carpal extensor muscles [40] were conducted on only three horses. Similarly, studies investigating the effects of trotting speed on muscle activity and kinematics were carried out on just four horses of two breeds [17], and five horses of two breeds [16]. Other studies, that included six [22], seven [11,27], or more horses [6,8,9,15,29], may be considered as beneficial. One may observe that in this study, the use of a uniform group of horses and the type of comparisons made—within ROI (and consequently within horse and gait), and across different variations of the exact same signal—justified the omission of normalization in the raw signal preprocessing protocol. The raw signal was preprocessed using DC–offset removal and notch filtering, without additional normalization. However, it is important to emphasize that when comparisons are made across horses, trials, strides, or gaits, signal normalization become a necessary preprocessing step to minimize individual variability in the dataset and ensure meaningful results [28].

Furthermore, the signal processing and analysis methods used in this study have certain limitations arising from differences in protocols compared to previous research, as well as the subjective aspects of signal processing—notably, the manual annotation of activity bursts and noise segments. Manual annotation introduces potential bias due to the subjective nature of ROI selection; however, this was mitigated through a clearly described annotation protocol to ensure a systematic approach. In this study, the annotation protocol used by Smit et al. [14] was followed, as it includes both burst and noise segments, which is essential for calculating the SNR. Nevertheless, unlike Smit et al. [14], this study did not include kinematic measurements, which represents another limitation. These limitations could be addressed by the development and application of automatic burst activity detection tools. However, such tools have not yet been developed specifically for equine sEMG signal processing. We believe that creating such tools represents a key direction for future research. This advancement could significantly enhance equine sEMG studies and broaden their applications in daily training, disease diagnosis, and the monitoring of rehabilitation and treatment programs.

## 5. Conclusions

The currently recommended standard for equine sEMG signal processing involves high–pass filtering with a cut–off frequency of 40 Hz, which is supported by the results of this study. Given the similar signal characteristic and the absence of differences in signal loss between high–pass filtering at 40 Hz and bandpass filtering at 40–450 Hz, results from studies using these two methods and similar designs appear to be comparable. Although both filtering methods result in the highest signal loss and corresponding filtering–induced changes in signal features, they also provide complete attenuation of the low–frequency noise component (0–20 Hz) during walk and trot, and nearly complete attenuation during canter. In contrast, all other noise attenuation filtering methods examined in this study resulted in only partial attenuation of this noise component, as well as varying levels of signal loss and associated changes in signal features. These filtering–induced changes should be carefully considered when comparing signal features from studies using different filtering approaches. The findings of this study may support cross-referencing in equine sEMG research and emphasize the need to apply standard filtering methods, particularly in studies related to training, rehabilitation programs, and the diagnosis of musculoskeletal disorders in horses.

## Figures and Tables

**Table 1:** Signal features (median and range (Q1; Q3)) of nine variations (raw and filtered signals) of the same sEMG signal recorded from the m. quadriceps femoris during walk. The data are presented across n = 6 horses.

	Amplitude [mV]	RMS [mV]	iEMG [mV×s]	MF [Hz]	SNR [dB]
raw signal	206.2 (170.0; 257.1)	46.4 (40.9; 54.1)	33.0 (29.4; 38.2)	48.8 (44.1; 54.4)	12.1 (10.8; 13.7)
low–pass 10 Hz	67.1 * (61.6; 84.2)	37.4 * (32.8; 43.6)	33.1 (29.3; 38.1)	2.9 * (2.7; 3.1)	12.5 (10.7; 13.9)
high–pass 20 Hz	197.2 (170.2; 240.8)	43.3 * (38.2; 50.6)	30.4 * (26.9; 34.9)	57.0 * (52.0; 62.5)	13.5 * (12.6; 14.8)
high–pass 40 Hz	173.8 * (149.6; 210.2)	33.4 * (30.9; 41.2)	22.7 * (20.8; 27.5)	80.7 * (74.2; 88.1)	13.6 * (12.4; 14.8)
bandpass 20–450 Hz	196.9 (169.7; 241.1)	43.3 * (38.2; 50.5)	30.4 * (26.9; 34.9)	56.9 * (52.0; 62.4)	13.5 * (12.6; 14.8)
bandpass 40–450 Hz	172.2 * (150.3; 212.8)	33.4 * (30.8; 41.1)	22.7 * (20.8; 27.5)	80.6 * (74.1; 87.9)	13.6 * (12.5; 14.8)
bandpass 7–200 Hz	197.5 * (166.5; 246.6)	45.4 (40.4; 53.2)	32.4 * (28.9; 37.5)	47.5 (43.1; 51.9)	12.2 (11.1; 13.9)
bandpass 15–500 Hz	198.5 (168.1; 256.3)	45.0 * (39.5; 52.6)	31.7 * (28.0; 36.3)	52.6 (47.4; 58.3)	13.3 * (12.0; 14.6)
bandpass 30–500 Hz	188.2 * (163.0; 224.8)	38.3 * (34.2; 45.4)	26.2 * (24.3; 21.4)	68.5 * (63.5; 74.7)	13.8 * (12.5; 15.0)

Signal features: amplitude, root mean square (RMS), integrated electromyography (iEMG), median frequency (MF), signal–to–noise ratio (SNR). Low-pass 10 Hz signal was compared separately. Asterisks (*) indicate differences in signal features between raw signal and filtered signals. Statistical significance was set at *p* < 0.05.

**Table 2:** Signal features (median and range (Q1; Q3)) of nine variations (raw and filtered signals) of the same sEMG signal recorded from the m. quadriceps femoris during trot. The data are presented across n = 6 horses.

	Amplitude [mV]	RMS [mV]	iEMG [mV×s]	MF [Hz]	SNR [dB]
raw signal	429.6 (373.0; 515.8)	114.5 (103.3; 130.8)	84.2 (77.8; 98.4)	51.8 (46.7; 55.7)	16.1 (14.4; 17.9)
low-pass 10 Hz	140.6 * (121.7; 168.1)	90.5 * (82.3; 103.0)	84.0 (77.5; 98.4)	3.9 * (3.6; 4.2)	15.3 * (13.8; 17.1)
high–pass 20 Hz	420.2 (357.3; 531.5)	108.9 * (95.4; 123.3)	79.0 * (72.4; 91.6)	57.7 * (52.8; 63.9)	17.0 * (14.5; 19.1)
high–pass 40 Hz	383.1 * (2295.2; 449.2)	86.8 * (78.7; 96.6)	63.9 * (57.2; 71.2)	77.7 * (70.9; 81.8)	16.7 * (14.8; 19.2)
bandpass 20–450 Hz	420.4 (356.7; 531.4)	108.9 * (95.3; 123.2)	79.0 * (72.3; 91.6)	57.7 * (52.8; 63.8)	17.0 * (14.5; 19.1)
bandpass 40–450 Hz	383.2 * (294.9; 450.2)	86.7 * (78.7; 96.6)	63.9 * (57.1; 71.1)	77.7 * (70.9; 81.1)	16.7 * (14.8; 19.2)
bandpass 7–200 Hz	417.9 (354.6; 505.9)	112.8 * (101.6; 129.2)	83.5 (76.9; 97.6)	50.9 (45.6; 54.8)	16.4 (14.4; 18.0)
bandpass 15–500 Hz	421.5 (362.0; 507.0)	112.2 * (99.8; 128.5)	82.0 * (75.1; 95.1)	55.0 (48.4; 58.7)	16.7 * (14.6; 18.8)
bandpass 30–500 Hz	405.2 * (318.4; 483.3)	98.4 * (86.5; 109.3)	71.4 * (64.6; 81.6)	66.5 * (61.3; 72.0)	16.8 * (14.6; 19.0)

Signal features: amplitude, root mean square (RMS), integrated electromyography (iEMG), median frequency (MF), signal–to–noise ratio (SNR). Low-pass 10 Hz signal was compared separately. Asterisks (*) indicate differences in signal features between raw signal and filtered signals. Statistical significance was set at *p* < 0.05.

**Table 3:** Signal features (median and range (Q1; Q3)) of nine variations (raw and filtered signals) of the same sEMG signal recorded from the m. quadriceps femoris during canter. The data are presented across n = 6 horses.

	Amplitude [mV]	RMS [mV]	iEMG [mV×s]	MF [Hz]	SNR [dB]
raw signal	1223.0 (1056.0; 1487.0)	353.8 (322.8; 402.4)	262.0 (233.3; 293.8)	61.0 (55.3; 69.1)	18.1 (16.2; 19.2)
low-pass 10 Hz	425.5 * (371.8; 487.0)	286.4 * (248.9; 317.1)	261.9 (231.1; 293.8)	8.5 * (8.2; 9.1)	17.0 * (15.1; 18.7)
high-pass 20 Hz	1203.0 (975.2; 1454.0)	337.0 * (305.2; 388.9)	250.8 * (221.9; 282.0)	64.9 * (59.8; 72.8)	18.3 * (16.2; 19.9)
high-pass 40 Hz	1029.0 * (859.0; 1330.0)	293.3 * (245.0; 333.3)	211.0 * (187.2; 245.1)	81.1 * (75.8; 86.6)	18.2 * (16.3; 20.2)
bandpass 20–450 Hz	1204.0 (967.1; 1451.0)	337.0 * (305.1; 388.8)	250.7 * (221.8; 282.0)	64.9 * (59.8; 72.8)	18.3 * (16.2; 19.9)
bandpass 40–450 Hz	1030.0 * (851.1; 1328.0)	293.1 * (245.0; 333.3)	210.8 * (187.1; 245.0)	81.0 * (75.8; 86.6)	18.2 * (16.3; 20.2)
bandpass 7–200 Hz	1134.0 * (997.9; 137.0)	347.8 * (315.2; 397.8)	258,4 * (229.3; 290.6)	60.3 (53.9; 67.3)	18.2 (16.1; 19.3)
bandpass 15–500 Hz	1232.0 (986.2; 1515.0)	348.9 * (315.0; 396.3)	259.2 * (227.7; 289.0)	62.8 (57.8; 71.4)	18.4 * (16.2; 19.9)
bandpass 30–500 Hz	1124.0 * (921.8; 1376.0)	313.8 * (282.3; 362.6)	231.4 * (204.3; 264.6)	72.5 * (66.7; 79.6)	18.0 * (15.8; 19.9)

Signal features: amplitude, root mean square (RMS), integrated electromyography (iEMG), median frequency (MF), signal-to-noise ratio (SNR). Low-pass 10 Hz signal was compared separately. Asterisks (*) indicate differences in signal features between raw signal and filtered signals. Statistical significance was set at *p* < 0.05.

**Table 4:** Signal loss metrics and power spectral density (PSD) (median and range (Q1; Q3) of eight variations (filtered signals in relation to raw signal) of the same sEMG signal recorded from the m. quadriceps femoris during walk. The data are presented across n = 6 horses.

	raw signal	low–pass 10 Hz	high–pass 20 Hz	high–pass 40 Hz	bandpass 20–450 Hz	bandpass 40–450 Hz	bandpass 7–200 Hz	bandpass 15–500 Hz	bandpass 30–500 Hz
signal loss	no applicable	19.8% (18.5; 20.8)	7.3% ^a^ (6.0; 8.7)	27.0% ^b^ (24.9; 29.8)	7.3% ^a^ (6.1; 8.8)	27.1% ^b^ (25.0; 29.9)	2.5% ^c^ (2.2; 2.9)	4.0% ^d^ (2.8; 5.1)	16.9% ^b^ (15.0; 19.1)
residual signal	no applicable	80.3% (79.2; 81.5)	92.8% ^a^ (91.4; 94.0)	73.0% ^b^ (70.2; 75.1)	92.7% ^a^ (91.3; 93.9)	72.9% ^b^ (70.1; 75.1)	97.5% ^c^ (97.1; 97.8)	96.1% ^d^ (94.9; 97.3)	83.1% ^b^ (80.9; 85.1)
PSD [mV^2^/Hz]	36.8 ^a^ (28.3; 60.0)	114.5 * (87.2; 158.6)	31.5 ^b^ (24.0; 51.8)	18.8 ^c^ (13.6; 26.5)	31.5 ^b^ (24.0; 51.8)	18.8 ^c^ (13.6; 26.5)	36.8 ^a^ (28.2; 60.0)	37.0 ^a^ (26.3; 55.6)	23.6 ^d^ (18.1; 35.7)

Signal loss metrics: signal loss, residual signal. Superscripts letters (a–d) indicate differences in signal loss metrics and PSD across signal variations except low–pass filtering at 10 Hz. Asterisks (*) indicate differences in PSD between raw signal and signal after the low–pass filtering at 10 Hz. Statistical significance was set at *p* < 0.05.

**Table 5:** Signal loss metrics and power spectral density (PSD) (median and range (Q1; Q3) of eight variations (filtered signals in relation to raw signal) of the same sEMG signal recorded from the m. quadriceps femoris during trot. The data are presented across n = 6 horses.

	raw signal	low–pass 10 Hz	high–pass 20 Hz	high–pass 40 Hz	bandpass 20–450 Hz	bandpass 40–450 Hz	bandpass 7–200 Hz	bandpass 15–500 Hz	bandpass 30–500 Hz
signal loss	no applicable	19.5% (18.6; 21.2)	6.3% ^a^ (4.9; 7.5)	25.2% ^b^ (22.1; 27.6)	6.4% ^a^ (4.9; 7.6)	25.3% ^b^ (22.1; 27.6)	1.5% ^c^ (1.2; 1.7)	2.7% ^c^ (1.9; 3.4)	15.6% ^d^ (13.7; 17.9)
residual signal	no applicable	80.5% (78.8; 81.4)	93.8% ^a^ (92.5; 95.1)	74.9% ^b^ (72.5; 78.0)	93.7% ^a^ (92.4; 95.1)	74.8% ^b^ (72.5; 77.9)	98.5% ^c^ (98.3; 98.8)	97.3% ^c^ (96.6; 98.1)	84.4% ^d^ (82.2; 86.3)
PSD [mV^2^/Hz]	200.2 ^a^ (160.9; 259.6)	435.3 * (361.2; 569.5)	175.8 ^b^ (142.0; 234.9)	118.1 ^c^ (85.8; 154.6)	175.8 ^b^ (142.0; 234.9)	118.1 ^c^ (85.8; 154.6)	199.8 ^a^ (161.2; 259.2)	191.7 ^ab^ (153.7; 254.7)	153.5 ^d^ (116.8; 185.9)

Signal loss metrics: signal loss, residual signal. Superscripts letters (a–d) indicate differences in signal loss metrics and PSD across signal variations except low–pass filtering at 10 Hz. Asterisks (*) indicate differences in PSD between raw signal and signal after the low–pass filtering at 10 Hz. Statistical significance was set at *p* < 0.05.

**Table 6:** Signal loss metrics and power spectral density (PSD) (median and range (Q1; Q3) of eight variations (filtered signals in relation to raw signal) of the same sEMG signal recorded from the m. quadriceps femoris during canter. The data are presented across n = 6 horses.

	raw signal	low–pass 10 Hz	high–pass 20 Hz	high–pass 40 Hz	bandpass 20–450 Hz	bandpass 40–450 Hz	bandpass 7–200 Hz	bandpass 15–500 Hz	bandpass 30–500 Hz
signal loss	no applicable	20.9% (18.3; 22.6)	3.7% ^a^ (2.3; 5.3)	17.0% ^b^ (14.6; 21.9)	3.7% ^a^ (2.4; 5.3)	17.0% ^b^ (14.6; 21.9)	1.5% ^c^ (1.0; 2.3)	1.6% ^c^ (0.9; 2.3)	9.9% ^d^ (7.2; 13.3)
residual signal	no applicable	79.1% (77.4; 81.7)	96.4% ^a^ (94.7; 97.8)	83.1% ^b^ (78.2; 85.5)	96.4% ^a^ (94.7; 97.7)	83.1% ^b^ (78.2; 85.5)	98.5% ^c^ (97.7; 99.0)	98.4% ^c^ (97.7; 99.1)	90.1% ^d^ (86.7; 92.8)
PSD [mV^2^/Hz]	1417 ^a^ (1075; 1889)	2274 * (1725; 2827)	1379 ^b^ (954; 1849)	1034 ^c^ (756; 1352)	1379 ^b^ (954; 1849)	1034 ^c^ (756; 1352)	1416 ^a^ (1070; 1886)	1404 ^a^ (1024; 1880)	1200 ^d^ (860; 1594)

Signal loss metrics: signal loss, residual signal. Superscripts letters (a–d) indicate differences in signal loss metrics and PSD across signal variations except low–pass filtering at 10 Hz. Asterisks (*) indicate differences in PSD between raw signal and signal after the low–pass filtering at 10 Hz. Statistical significance was set at *p* < 0.05.

## Data Availability

The data presented in this study are available upon request from the corresponding author.
